# A Microfluidic High-Capacity Screening Platform for Neurological Disorders

**DOI:** 10.1021/acschemneuro.3c00409

**Published:** 2023-12-27

**Authors:** Lydia Moll, Johan Pihl, Mattias Karlsson, Paul Karila, Camilla I. Svensson

**Affiliations:** † Cellectricon AB, Mölndal 431 53, Sweden; ‡ Department of Physiology and Pharmacology, Center for Molecular Medicine, Karolinska Institutet, Stockholm 171 76, Sweden

**Keywords:** microfluidics, neurological disorders, drug discovery, plate-based screening, electrophysiology, in vitro systems

## Abstract

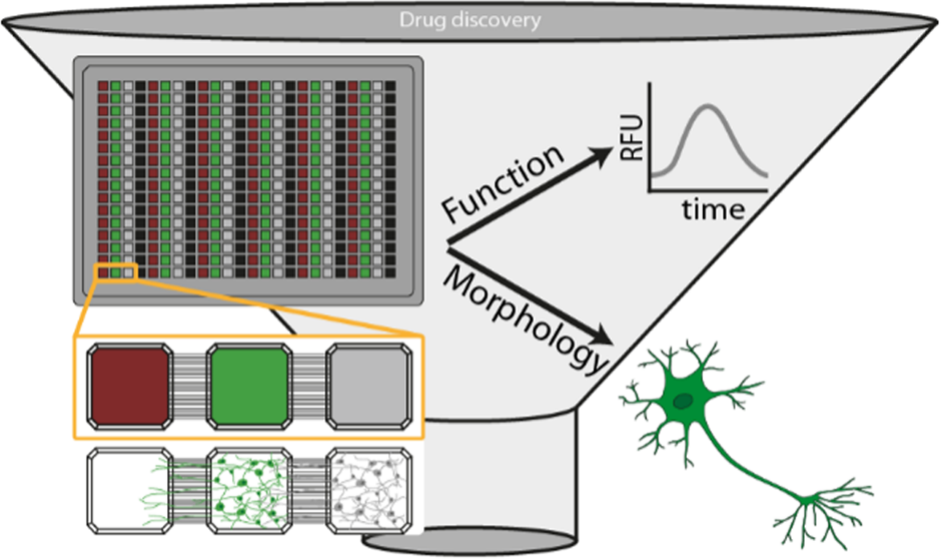

Compartmentalized cell cultures (CCCs) provide the possibility to study mechanisms of neurodegenerative diseases, such as spreading of misfolded proteins in Alzheimer’s or Parkinson’s disease or functional changes in, e.g., chronic pain, in vitro. However, many CCC devices do not provide the necessary capacity for identifying novel mechanisms, targets, or drugs in a drug discovery context. Here, we present a high-capacity cell culture microtiter microfluidic plate compliant with American National Standard Institute of the Society for Laboratory Automation and Screening (ANSI/SLAS) standards that allows to parallelize up to 96 CCCs/experimental units, where each experimental unit comprises three microchannel-connected compartments. The plate design allows the specific treatment of cells in individual compartments through the application of a fluidic barrier. Moreover, the compatibility of the plate with neuronal cultures was confirmed with rodent primary as well as human-induced pluripotent stem cell-derived neurons of the central or peripheral nervous system for up to 14 days in culture. Using immunocytochemistry, we demonstrated that the plate design restricts neuronal soma to individual compartments, while axons, but not dendrites, can grow through the connecting microchannels to neighboring compartments. In addition, we show that neurons are spontaneously active and, as deemed by the appearance of synchronous depolarizations in neighboring compartments, are synaptically coupled. In summary, the design of the microfluidic plate allows for both morphological and functional studies of neurological in vitro cultures with increased capacity to support identification of novel mechanisms, targets, or drugs.

Modeling aspects of neurological diseases (NDs), such as Alzheimer’s disease (AD), Parkinson’s disease (PD), amyotrophic lateral sclerosis (ALS), and chronic pain in vitro, is challenging. Compartmentalized cell cultures (CCCs) present a promising approach for advancing in vitro research on new mechanisms and targets in the quest to develop new drugs and alleviate NDs. However, the current limitations on available devices, including their low capacity, hinder their applicability in high-throughput drug discovery.^1,2^

In CCCs, discrete cell culture regions are connected via microchannels with diameters large enough to establish a fluidic connection between the regions but sufficiently small to restrict cell migration. This enables spatial and temporal separation of cellular regions (such as soma and axons) or different cell populations while allowing cellular communication between these.^3^

The use of CCCs allows for the study of various key mechanisms of NDs including the uptake, aggregation, axonal transport, and cell-to-cell spreading of misfolded proteins.^4−6^ Additionally, CCCs are employed to investigate axonal degeneration and regeneration,^7,8^ as well as the transport of proteins, ion channels, and organelles along axons.^9,10^ Furthermore, CCCs can be utilized to evaluate functional aspects such as neuronal excitability and cellular communication between distinct neuronal populations or between neurons and non-neuronal cells^11,12^ (for extensive reviews on CCC applications in NDs, see refs (3,13−15)).

Currently used CCC devices frequently have complex designs that come with the trade-off of limited capacity. While they are primarily utilized for basic research applications, the limited capacity is a barrier to employing these CCC models in target and drug screening contexts.^2^ Therefore, increased capacity is necessary, not only through the scale-up and parallelization of CCC devices but also by considering compatibility with robotic liquid handling and laboratory automation.^1^ Moreover, it requires robust manufacturing and materials to ensure reliable and reproducible results.^1^

In recent years, different microfabrication techniques have been employed to produce substrates for CCC formation. The most common method is soft lithography using replica molding to produce poly(dimethylsiloxane) (PDMS) devices.^16^ Although these PDMS devices are inexpensive, easy to manufacture and prototype, and highly cell-compatible,^16^ they also have certain limitations. For instance, PDMS is hydrophobic, which makes it challenging to fill the structures with liquids, and therefore the devices are prone to bubble formation and absorbance of hydrophobic drugs.^17^ Furthermore, the devices can leak non-cross-linked material and are instable to many organic chemicals,^17^ which limits their drug screening potential. Alternative materials that are widely used in cell culture include hard plastic materials such as polystyrene (PS) or cyclic olefin copolymer (COC) or glass. These materials are frequently used in microtiter plates in screening applications and have also been used in approaches to scale up microfluidic models.^18,19^ Nonetheless, generating microscale structures, such as microchannels, that are small enough to prevent neuron migration while allowing for cellular communication between cell compartments and ensuring reliable fabrication with tight tolerances is challenging to achieve in hard plastic materials.

Here, we introduce a novel cell culture hard plastic-based microtiter microfluidic plate (MC-plate) (European patent application EP3892713A1) specifically designed for studying the mechanisms of NDs. The MC-plate offers a high-density array of CCCs and complies with the American National Standard Institute of the Society for Laboratory Automation and Screening (ANSI/SLAS) standards. The plate design and channel dimensions restrict neuronal soma to individual compartments, while axons, but not dendrites, can grow through the connecting microchannels to neighboring compartments.

In summary, through compatibility with laboratory automation and parallelization, this plate-based CCC provides sufficient capacity to be amenable to the screening of novel targets and drugs.

## Results and Discussion

### Design and Characterization of the Microchannel Microtiter Plate (MC-PLATE)

#### Technical Development and Manufacturing

**Figure 1:**
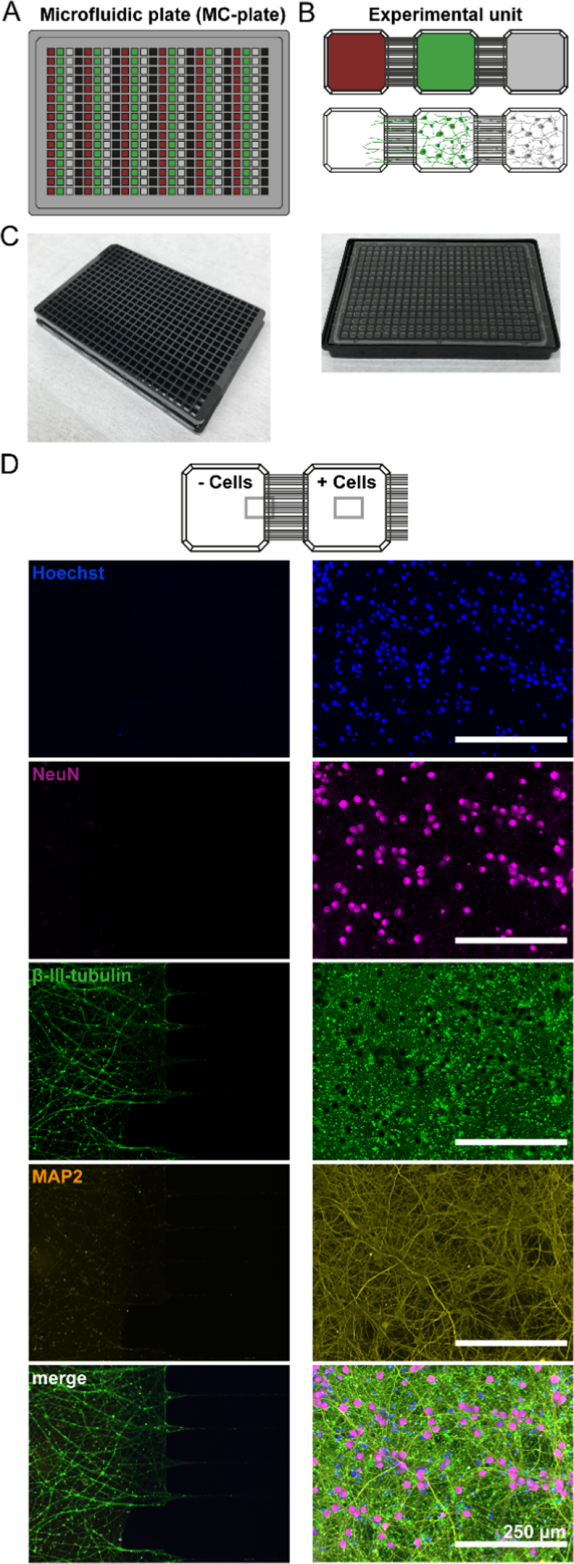
(A) Schematic drawing of the 96 experimental units of the microchannel plate. (B) Three individual compartments and microchannel connections of one experimental unit, including a possible cell layout. (C) Photographic pictures of the plate top and bottom. (D) Immunocytochemical staining of mouse cortex cultures plated in the center compartment at 14 DIV. Images show an excerpt of compartment 1 (close to the channels) and compartment 2 (middle) of β-III-tubulin (green) and MAP2 (yellow), NeuN (magenta), and Hoechst (blue). Microscope information: ImageXpress Confocal HT.ai (Molecular devices), 20× magnification.

To address the scalability challenges of CCCs, we developed a design based on the standard 384-well plate layout, complying with the ANSI/SLAS standard (Figure 1A). The plate consists of 384 individual compartments of which up to four compartments are connected forming a CCC-experimental unit. In the experiments presented herein, excluding the functional experiments, we used a plate with three open compartments connected by microchannels, resulting in 96 experimental units per plate (Figure 1A,B). This design provides the necessary capacity and laboratory automation compatibility for screening scenarios in target discovery and drug development.^1,2^ The MC-plate is composed of three layers of hard plastic: a black COC top layer in a standard 384-well plate format (Figure 1C, left), a clear microfluidic middle layer, and a clear bottom film to seal the plate (Figure 1C, right). COC was chosen because it is a commonly used material in microtiter plates for drug screening applications, and has been shown to be biocompatible, resistant to water-induced swelling, drug-compatible, and offers superior imaging clarity compared to other cell culture plastics such as polystyrene.^20^ The individual layers were assembled by thermal fusion bonding,^20^ thereby eliminating the potential adverse effects from adhesives. Neuronal cell cultures in the fusion-bonded MC-plates were evenly distributed throughout the compartment (Supplementary Figure S1), indicating that the bonding method did not have any negative impact.

Producing and bonding microchannels with small dimensions in hard plastic materials is a challenging task. Hot embossing has previously been described as a method to fabricate microfluidic hard plastic devices based on COC^20,21^ or poly(methyl methacrylate) (PMMA).^22^ We investigated the feasibility of hot embossing a microfluidic COC layer with different channel designs including alterations of channel widths (5, 10, 14, and 30 μm), heights (5, 10, and 14 μm), and lengths (900 and 1000 μm) to produce the MC-plate. Channels with a height of 5 μm at all widths exhibited frequent structural failure upon bonding and were challenging to fill using vacuum filling (assessed qualitatively with light microscopy). In contrast, channels with 10 and 14 μm heights bonded without structural failure, allowing for serial production with established microfabrication methods, and were straightforward to fill using vacuum filling. Our cell-based experiments showed that channel dimensions of 5 × 10 μm (W × H) prevented neuronal migration, as assessed by immunocytochemical labeling with the neuronal marker NeuN^23^ in primary mouse cortex cells cultured for 14 days in vitro (DIV) (Figure 1D). The experimental setup involved an overall channel length of 900 μm, which was used to separate axons from dendrites over extended periods of time (Figure 1D). Previous work by Taylor et al.^8^ demonstrated that dendrites of rodent central neurons did not grow through channels with lengths greater than 450 μm at 14 DIV, while axons reached channel distances of 900 μm. To confirm this separation, mouse cortex neurons were cultured for 14 DIV in the central compartment, and expression of the dendrite-specific marker MAP2^24,25^ visualized by immunocytochemistry. Intense MAP2 positive staining was observed in the central compartment while low intensities of MAP2, colocalizing with the neuronal cytoskeleton marker β-III-tubulin,^26^ were present in the neighboring, empty compartment. Positive MAP2 labeling in axons, colocalizing with tau, has previously been reported in cortex cultures in vitro.^24^ We therefore concluded, together with the tau-staining presented in Figure 3C, that dendritic growth was restricted to the central compartment, while we observed an extensive growth of axons through the microchannels to the empty neighboring compartment.

Besides supporting physical separation of axons from cell soma and dendrites, a channel length of 900 μm was selected due to manufacturability aspects as it allowed the inclusion of expanding funnels at the channel endings and an expanded higher box (Supplementary Figure S2). Both features support the alignment during the milling of the compartment troughs and reduce the risk of debris from the milling procedure clogging the microchannels.

**Figure 2:**
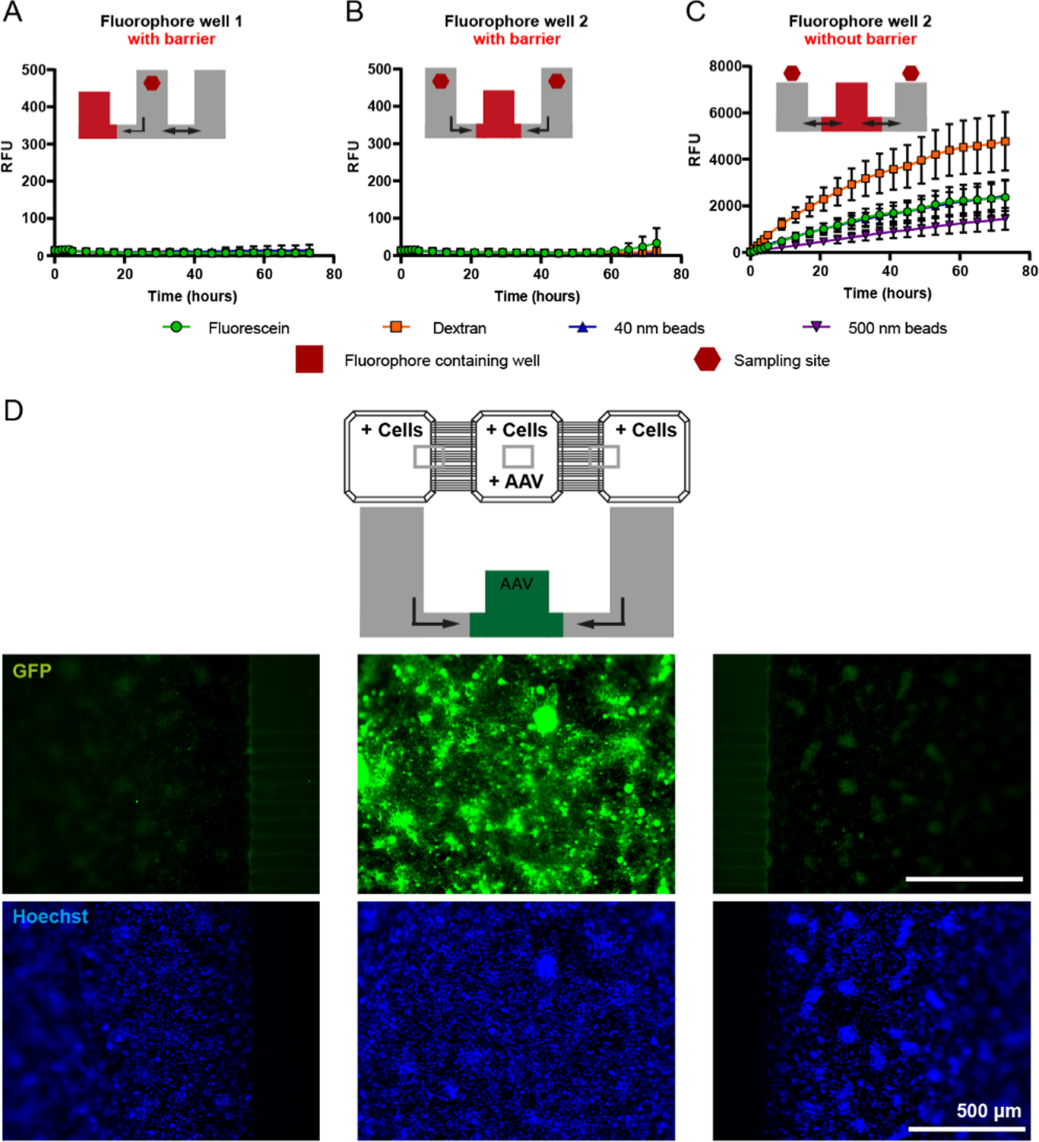
(A–C) Fluidic isolation by the establishment of a hydrodynamic barrier retains fluorescent substances (red compartment) within the compartments for more than 70 h, while free diffusion is observed without a hydrodynamic barrier. Fluorescent intensity was monitored in compartment 2 with fluorophore in compartment 1, alternatively in compartments 1 and 3 with fluorophore in the center compartment as indicated by the hexagon in the schematic. In addition, for each graph (A–C) liquid levels and flow directions are indicated in the schematic drawing. Graphs are represented as mean ± SD (D) Mouse cortex cells seeded in all 3 compartments of an experimental unit. AAV8 shRNA with GFP reporter was added to the center compartment with an established hydrodynamic barrier (compare schematic drawings). While cells are visible by Hoechst-positive nuclei (blue) in all three compartments, GFP (green) fluorescence is retained in the center compartment. Microscope information: Operetta (PerkinElmer), 10× magnification.

To maximize the possibility of axons growing to neighboring compartments without compromising fluidic isolation between the compartments, the number of connecting channels was varied between 1, 10, or 30. It was noted that 30 channels provided good fluidic isolation (Figure 2). The 30-channel design was thus chosen to maximize the interaction between axons, which grow through the microchannels, and cells or compounds added to the adjacent compartment.

#### Fluidic Retention of Content in Discrete Compartments Enables Spatially Defined Drug Targeting and Assessment of Neurobiological Mechanisms

To achieve the isolated treatment of individual compartments, a hydrodynamic barrier was established to prevent diffusion through the microfluidic channels. This was achieved by doubling the fluid volume from 50 to 100 μL in the adjacent compartments compared to the treated compartments, resulting in a liquid level difference of approximately 5 mm (the slight increase in width at the top of the plate was ignored during the calculations) and a pressure difference of 49.5 Pa (with ρ = 1000 kg/m^2^, *g* = 9.81 m/s^2^). This volume difference directed the flow from the adjacent compartments into the treated compartments and constrains the treatment to individual compartments. The method of elevated liquid pillars has previously been applied to separate the treatments of distinct compartments. For example, Taylor et al.^8^ applied a hydrodynamic barrier to investigate the effects of BDNF and NT-3 treatment on isolated axons in axotomized cultures. While the MC-plate is a nonperfused device, a similar approach of hydrostatic pressure-directed flow is applied in other microfluidic-compartmentalized structures to induce perfusion of, e.g., cell cultures and lab-on-a-chip devices.^28^ Fluorescent molecules and beads were utilized to investigate the integrity of treatments with and without a hydrodynamic barrier in the MC-plates. Applying the elevated liquid pillar in the adjacent compartments restricted the diffusion of fluorescein, 70 kDa dextran, and fluorescent beads of different sizes (40 or 500 nm), as assessed by monitoring the fluorescent intensity in neighboring compartments, for up to 72 h (Figure 2A,B). Without this gradient, free diffusion through the microchannels was observed within the first 10 h after application (Figure 2C). To confirm the isolated, targeted treatment in a cell culture environment, mouse cortex cultures were established in all three compartments for 10 days. Subsequently, a hydrodynamic barrier was established in the adjacent two compartments by doubling the fluid volumes and an adenovirus scrambled shRNA with a GFP reporter was added to the center compartment. At 14 DIV, GFP expression was observed in the transfected cells in the center compartment, while the cell cultures in the neighboring compartments showed no fluorescence (Figure 2D; for non-AAV treated controls, see Supplementary Figure S3). This confirmed that the hydrodynamic barrier prevented diffusion of the virus into the adjacent compartments also in the presence of cells.

### Neuronal Cell Culture Compatibility

#### Cultures of Different Cell Types Can Be Maintained in the Experimental Units

**Figure 3:**
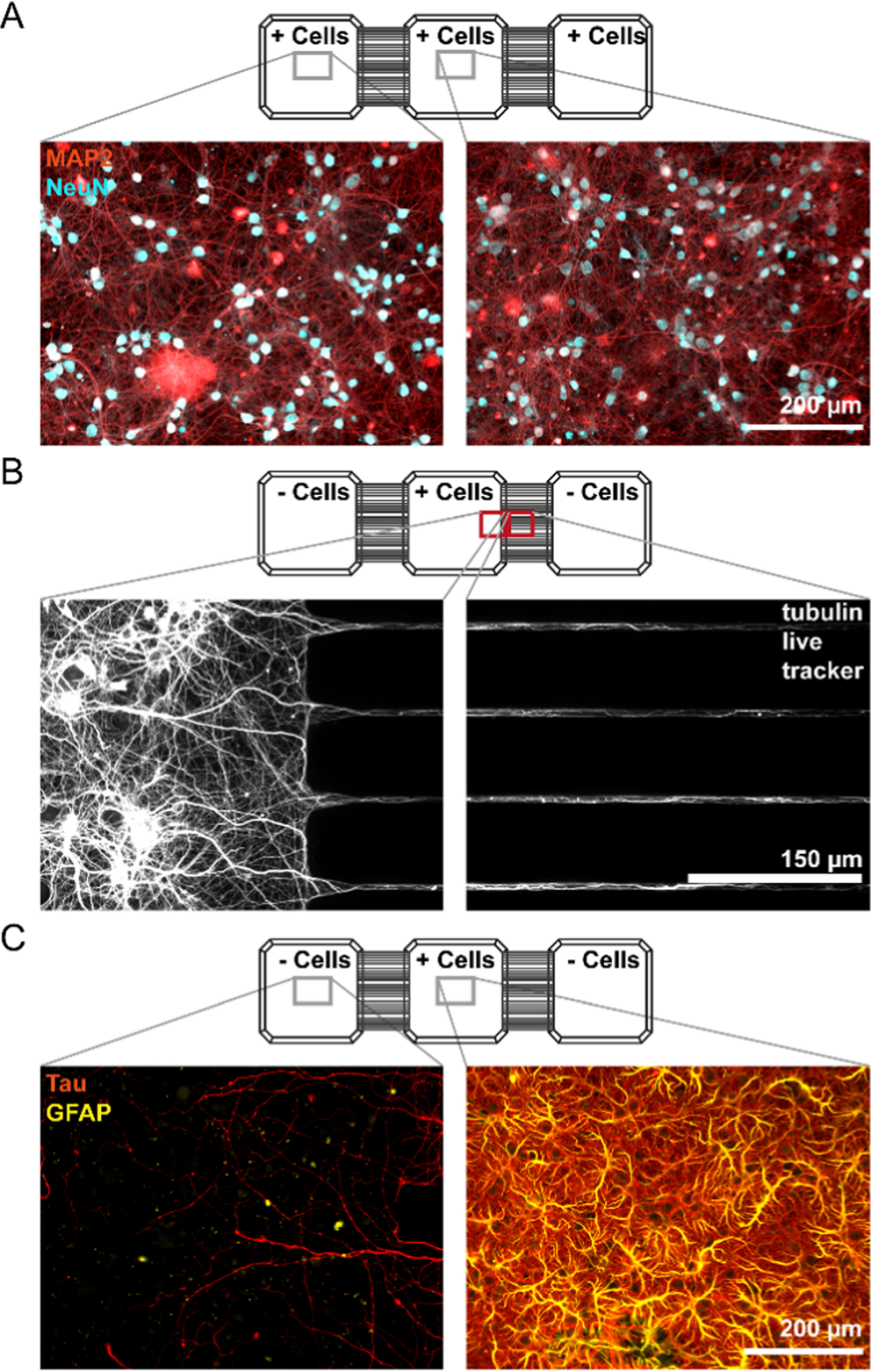
Representative immunocytochemical images of a single field of view of two compartments. The schematic drawing represents cell seeding layouts and approximate sites of imaging. Note that the schematic is not to scale. (A) Mouse cortex cells seeded in all 3 compartments fixed at 14 DIV, stained with the dendritic marker anti-MAP2 (red) and the neuronal marker anti-NeuN (cyan blue); (B) Mouse cortex cells seeded in the center compartment at 14 DIV, live imaged with the tubulin tracker Deep Red in the cell well and within channels. Brightness and contrast were adjusted for the cell well and channels individually; (C) Mouse cortex cells seeded in the center compartment fixed at 14 DIV, stained with the axonal marker tau (red) and the glia cell marker GFAP (yellow). Microscope information: Operetta (PerkinElmer), 20× magnification (A, C), ImageXpress Confocal HT.ai (Molecular devices), 40× magnification.

To allow the modeling of NDs, the MC-plate needs to support the growth and functionality of both neuronal and non-neuronal cells, including, for example, primary or induced pluripotent stem cell (iPSC)-derived CNS cultures and primary or iPSC-derived cultures of the peripheral nervous system. To ensure compatibility, the MC-plates were tested by seeding mouse cortex cultures in one or all three compartments (Supplementary Figure S4). When cortex cultures were seeded in all three compartments, an extensive neurite network was observed within all three compartments at 14 DIV (Figure 3A), as indicated by the dendritic marker MAP2.^24,25^ However, axonal crossing was not assessed in this setup. On the other hand, when mouse cortex cells were seeded in the center compartment only, and the adjacent compartments were left cell-free, tau immunoreactive axons^24,25^ were observed to grow into the adjacent compartments (Figure 3C).

Thus, this approach allows for the identification and isolation of axonal structures. In addition, this observation is consistent with the data presented in Figure 1D, where MAP2 immunoreactive dendrites and neurons were confined to the compartment where the neurons were seeded, while β-III-tubulin immunoreactivity, which is distributed throughout the neuronal cytoskeleton, including axons, was observed in adjacent empty compartments. The neuronal cells were evenly distributed, as indicated by NeuN immunoreactivity (Figure 3A), and the overall cellular morphology and survival appeared similar to cortex cultures in standard 384-well plates (Supplementary Figure S5). Although NeuN staining is predominantly associated with the nucleus, positive immunocytochemical staining and cytoplasmic localization of the protein has also been reported,^23^ which is in accordance with the staining pattern in our cultures. The compartmentalization of soma and axons enables investigation of localized effects, such as changes in excitability or morphology, after treatment on the axonal or soma level. Furthermore, microscopic evaluation of structures within the channels is possible, as exemplified by staining with tubulin tracker Deep Red (Figure 3B). This can be useful in the evaluation of transport processes within axons^6,10^ and axonal degeneration or regeneration processes.^29^

In addition to neurons, non-neuronal cells such as astrocytes (as indicated by immunoreactivity to GFAP^30^) were also present in the mouse cortex cultures in the MC-plates (Figure 3C) as well as in the standard 384-well cultures (Supplementary Figure S5).

**Figure 4:**
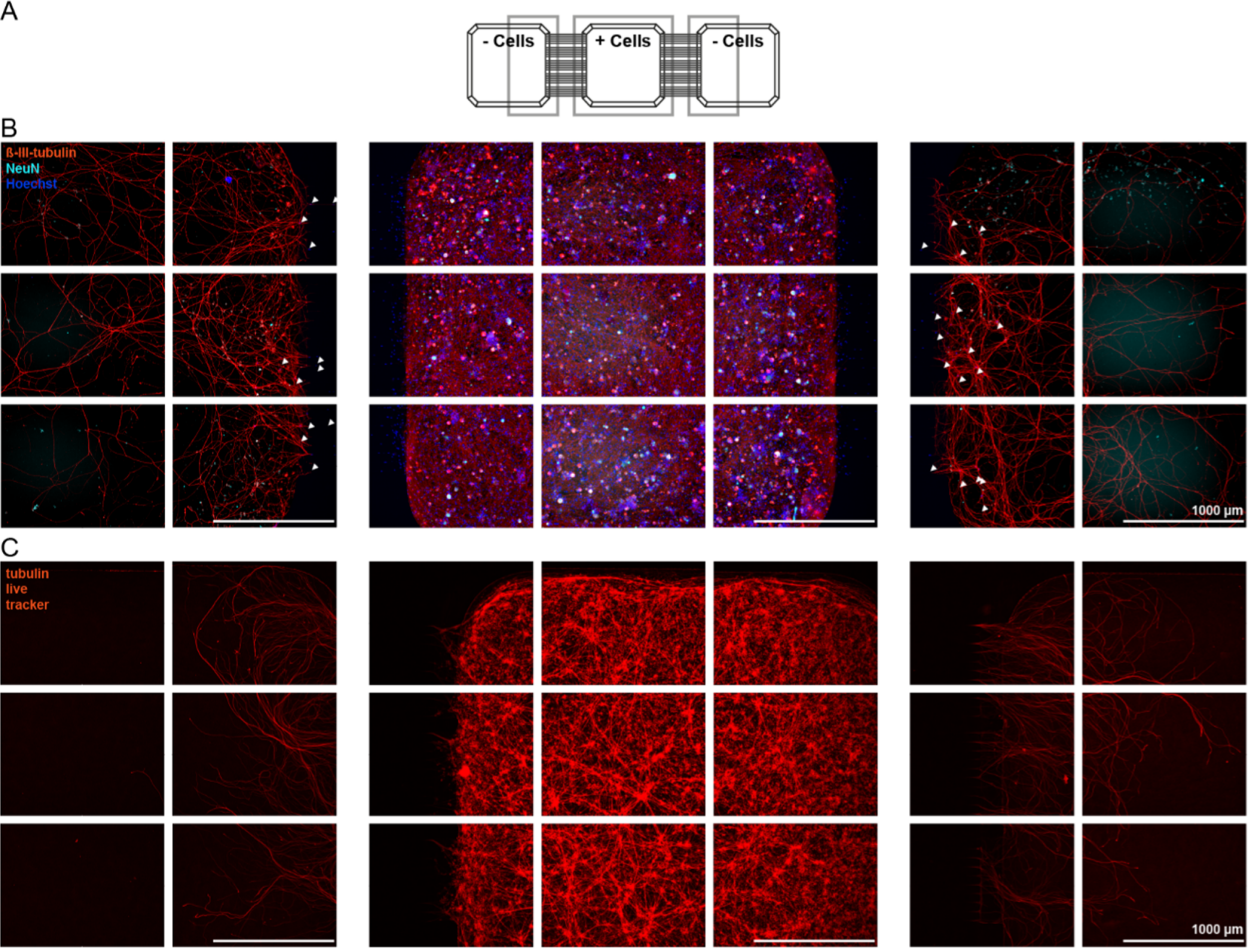
(A) Schematic drawing of the cell seeding setup and imaging areas corresponding to the displayed images below. Note that the schematic is not to scale. (B) Peripheral DRG neurons at 13 DIV seeded in one center compartment with axonal marker anti-β-III-tubulin (red) and neuronal marker NeuN (cyan blue) and nuclei marker Hoechst (blue), white arrowheads indicating nuclei of migrating cells. (C) Live staining with tubulin tracker Deep Red of iPSC-derived sensory neurons at 14 DIV after seeding in one center compartment of the MC-plate. Fluorescent background in the live image was subtracted with a rolling ball radius. For visualization brightness settings in the cell well and adjacent wells differ. Microscope information: Operetta (PerkinElmer), 10× magnification.

To also evaluate the compatibility of the MC-plate with primary cells from the peripheral nervous system, we seeded rodent dorsal root ganglia (DRG) neurons in the center compartment of an experimental unit (Figure 4A). The DRG neurons grew extensive axons both within the compartment and through the microchannels into adjacent compartments (Figure 4B). Our results on DRG neurons were in accordance with those obtained from CNS cortical cultures, as NeuN-positive neurons remained restricted to the compartment where they were seeded. However, in DRG cultures, we observed small Hoechst-positive nuclei along axons in the microchannels and in the empty compartments (Figure 4B), indicating cells having migrated through the microchannels. These migrating cells were most likely glial cells, such as satellite glial cells or Schwann cells, which are both inherent to the DRG. Especially satellite glia cells, which enwrap the DRG neuronal cell bodies in vivo, are known to move away from the neuronal cell bodies and distribute in culture over time.^31^ Similarly, Schwann cells are known to accompany axonal growth;^32^ therefore, a joint migration through the microchannels may be another explanation.

The possible presence of both glial cell types in the DRG cultures was confirmed by immunocytochemistry using the glial cell marker GFAP (Supplementary Figure S6), which is present in both satellite glial and Schwann cells.^31,32^ In addition, we also evaluated the compatibility of the MC-plate with human iPSC-derived sensory neurons by culturing them in the center compartment for up to 3 weeks. The iPSCs were allowed time for neuronal maturation and the formation of extensive processes that grew through the microchannels into the empty, adjacent compartments. To visualize axonal outgrowth in the iPSC-derived sensory neuronal cultures, we used a cellular tubulin live tracker at 14 DIV (Figure 4C).

#### Cultures Are Spontaneously Active and Connected between Microchannel-Separated Compartments

The assessment of neuronal function in CCCs is crucial in studying the communication between different neuronal cell types, such as investigating peripheral and central sensitization in chronic pain.^12^

**Figure 5:**
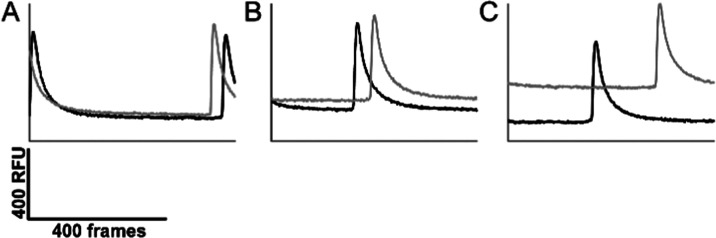
Representative traces of synchronicity. (A, B) The neuronal cultures are synaptically connected via the microchannels, as evidenced by the synchronous behavior of the calcium oscillations (as measured by the fluorescent calcium indicator Ca5). The calcium oscillations start randomly in either the right or left compartment. (C) Example of nonsynchronous behavior in an experimental unit; Legend: gray: left chamber; black: right chamber. RFU: relative fluorescent units.

Therefore, we tested the utility of the MC-plate for measuring neuronal function by seeding mouse cortex cells in two adjacent compartments, forming an experimental unit. The activity was recorded for 60 s at a frame rate of 10 Hz with an optical electrophysiology platform in combination with a calcium indicator.^33^ Recording of the fluorescence intensity of the calcium indicator showed the spontaneous activity of the cells in all compartments (Figure 5; Table 1). Additionally, 42% of the cultures in the microchannel-connected compartments displayed synchronized changes in fluorescence intensity (Table 1, Figure 5A–C), indicating that the cultures residing in separate compartments were coupled, likely through synaptic mechanisms. Moreover, the variability in the fluorescence baseline and peak signal was low between the evaluated compartments (Table 2).

**Table 1:** Quantification of Spontaneous and Synchronous Activity of Compartments and Units^a^

	*n*	%
compartments displaying spontaneous activity	24/24	100
units displaying spontaneous activity	12/12	100
units displaying synchronous activity	5/12	42

a (*n*) Number of assessed wells in one MC-plate.

**Table 2:** Variation in Baseline and Maximum Fluorescence Values^a^

	mean	SD	%CV	*n*
baseline RFU	998.3	65.3	6.5	24
maximum RFU	1492.4	87.3	5.8	24

a (*n*) Number of assessed wells in one MC-plate.

#### Limitations

To allow for increased capacity, compromises in the design of the plate-based CCC were necessary that led to reduced complexity compared with other CCC devices. For example, as for other standardized multiwell plates for high-throughput screening, the culture compartments are not perfused, which hinders quick or continuous fluid exchange across the cell cultures. Moreover, the MC-plate was evaluated by using 2D culturing conditions, while the feasibility of applying 3D hydrogel culturing approaches remains to be assessed.

## Conclusions

In summary, we here introduce a novel cell culture hard plastic-based, microtiter, microfluidic-compartmentalized plate, which holds potential for use in screening of targets and drugs for NDs. The broad compatibility with both primary and iPSC-derived neuronal cultures presents a unique opportunity for drug and target screening across different model systems. This approach not only addresses ethical considerations by reducing the reliance on in vivo experiments but also ensures the application of 3R principles in research. Moreover, while originally designed for applications within the field of neuroscience, the versatile design of the MC-plate can support other research fields such as immunology and oncology as well as the study of bidirectional interaction between neurons and other cell types. This adaptability highlights the potential for interdisciplinary applications, broadening the scope of the MC-plate beyond neuro-centered research.

## Materials and Methods

### Plate Manufacturing

Plates are manufactured in cleanroom facilities by Micronit.

### PLO-Laminin

Plates are coated with 0.01% Poly-l-ornithine (PLO; P3655, Sigma) for 24 h at room temperature (RT). After washing with sterile water, a coating of 10 μg/mL Laminin (L2020, Sigma) was applied for at least 1 h.

### Matrigel

Plates are coated with Matrigel (356230, Corning) in Neurobasal medium (A13712–01, Life Technologies) at a final concentration of 0.33 mg/mL for at least 1 h at 37 °C before cell addition.

### Animals

All experiments were conducted in accordance with European and Swedish animal welfare regulations under ethical permit 76–2013/188–2013/5.8.18–11305/2018/5.8.18–16036/2019/5.8.18–10841/2023. Animals were housed, and dissections were performed at the Department of Experimental Biomedicine, Gothenburg University.

### Mouse Cortex Cells

Female, pregnant C57bl6 mice were sedated and euthanized by cervical dislocation, and embryos were removed from the uteri and decapitated. The embryo brains were exposed, and cortices were removed. Cortices are transferred and triturated in Hibernate E wo Ca^2+^ (A1247601, Gibco). The cells were collected and seeded at densities of 20 000–50 000 cells/compartment in Neurobasal plus (A3582901, Gibco), supplemented with 0.1% Gentamycin (15710064, Gibco), 2% B27 Plus (A3582801, Gibco), 0.25% Glutamax (35050038, Gibco), or NbActive (NbActiv4500, BrainBits LLC) with 0.1% Gentamycin. All media was supplemented with 20 ng/mL NGF (556-NG-100/CF, R&D System) and 20 ng/mL BDNF (450–02, Preprotech). A 50% media exchange was performed once a week.

### Adult Rat DRGs

Adult DRGs, from 5- to 8-week-old male Sprague–Dawley rats (approximately 42–48 DRGs from sacral to cervical vertebrae), were microsurgically dissected. Ganglia were transferred to L-15 media (supplemented with 1% penicillin/streptomycin (PAA laboratories #P11–010), 24 mM NaHCO_3_ (Scharlab #SO0131), and 38 mM d-glucose (Sigma-Aldrich #G8769)), and nerve endings were trimmed under a stereomicroscope. Tissue was then enzymatically dissociated in L-15 medium for 1 h at 37 °C. The enzymatic reaction was stopped by adding 10% FBS (Hyclone #SV30160.03). The cells were mechanically dissociated by trituration with a fire-polished Pasteur pipet. After trituration remaining suspension was flushed through a 40 μL cell strainer (Falcon, Fisher Scientific). The cells were spun at 200x g for 5 min, and the pellet was resuspended in Neurobasal A Medium (Gibco #10888–022) supplemented with 1% penicillin/streptomycin, 1x Glutamax (Gibco #35050–038), 1x B27 supplements (Gibco #17504–044), and 5 ng/mL recombinant rat NGF (R&D Systems #556-NG-100CF). A 50% media exchange was performed every 2–3 days.

### iPSC

Neuroprogenitors for iPSC-derived sensory neurons were purchased from Censo Bio and seeded in Matrigel-coated plates at a density of 60 000 cells/compartment in N2B27 medium (A127122–01, Life Technologies), 2% B27 supplement without vitamin A (12587–010, Life Technologies), 1% N-2 supplement (17502–048, Life Technologies), 2% Glutamax (35050–038, Life Technologies), 0.06% Gentamycin (15750–045, Life Technologies), 100 nM 2-mercaptoethanol (Sigma-Aldrich), and 10 μM rock inhibitor Y26732. After 24 h, medium was replaced with fresh N2B27 medium supplemented with growth factors (GF): 25 ng/mL NGF (450–01, Peprotech), 25 ng/mL GDNF (450–10, Peprotech), 10 ng/mL NT3 (450–03, Peprotech), and 10 ng/mL BDNF (450–02, Peprotech). To suppress cell division on 3 DIV, 1 μg/mL Mitomycin C (M4287, Sigma) was added to the cells and replaced after 2 h with N2B27 with GF. Media was, subsequently, exchanged every 3–4 days with N2B27 with GF.

### Fluidic Isolation

Compartments were filled with 50 μL of either 5 μM fluorescein (F7137, Sigma), 62.5 μg/mL 70 kD fluorescein-labeled dextran (D1823, Invitrogen), or fluorescein-labeled beads with 40 nm (0.025%, F10720 Invitrogen), respectively, 500 nm diameter (0.008%, F8813, Invitrogen). Adjacent compartments were filled with either equal volumes or twice the volume to establish a direct flow. Plates were sealed and imaged for 72 h on an Infinite 500 plate reader (Tecan) at 485/535 nm (excitation/emission).

### AAV-Transduction

Mouse cortex cells at 10 DIV were transfected with pAAV8-U6-GFP-scrambled control (lot no. 2018.04.09, Vigene Biosciences, Inc.). At 14 DIV, cells were counterstained with Hoechst 33342 (H3570, Invitrogen) and imaged on a PerkinElmer Operetta high content imaging system with a 10× 0.4 NA objective.

### Functional Readout

The functional readout was performed on Cellectricon’s optical electrophysiology platform as previously described^33^ but with mouse cortex neurons. Neurons were stained with the fluorescent calcium indicator Calcium 5 (FLIPR calcium 5 assay kit R8185, Molecular Devices). Calcium 5 was resuspended in cell-specific medium, 10% added to the cell cultures, and incubated for 1 h at 37 °C, 5% CO_2_. Spontaneous depolarizations were recorded as a change of fluorescence intensity with an exposure of 80 ms and a frame rate of 10 Hz over 60 s.

### Tubulin Live Tracker

Tubulin tracker deep red (T34076, Invitrogen) was resuspended according to the manufacturer’s instructions and added to the iPSCs cells at 13 DIV or mouse cortex cultures at 14 DIV with a final working concentration of 1x in standard culture medium. The cells were incubated for 30 min at 37 °C with 5% CO_2_, and the staining solution was then replaced with cell-specific medium. The cells were imaged at the Operetta imaging system at 37 °C with 5% CO_2_ or the Image Xpress Confocal HT.ai (Molecular Devices).

### Immunocytochemistry

For immunocytochemistry, cell cultures were fixed for 20 min with 4% PFA. Subsequently, the cultures were blocked with 2% normal goat serum (31873, Fisher), and 0.2% Triton-X (T8787, Sigma) in PBS before cells were incubated with primary antibodies (Supplementary Table S1) overnight at 4 °C. On the next day, primary antibodies were removed, and plates were thoroughly washed and incubated with secondary antibodies (Supplementary Table S2) and Hoechst 33342 (H3570, Invitrogen) for 2 h at RT.

### Imaging

Plates were imaged on the Operetta high content imaging system (PerkinElmer) with either a 10x 0.4 NA, 20x 0.75 NA, 20x 0.45 NA objective in wide-field or confocal mode or with the Image Xpress Confocal HT.ai (Molecular Devices) with a 20x 0.95NA or 40x 1.15NA water immersion objective in confocal mode. Excitation/emission wavelengths are listed in Supplementary Table S3.

### Display

Graphs were prepared with GraphPad Prism 9.5.1, and illustrations were created with Adobe Illustrator. Images were adjusted for brightness and contrast equally over the experimental unit unless stated otherwise in the figure description and pseudo-colored in ImageJ (Fiji) for better visualization.

## Supporting Information Available

Antibodies, imaging information, and additional microscopy images (PDF)
