# Supplementary material for: A Microfluidic High-Capacity Screening Platform for Neurological Disorders
Source: ACS Chem Neurosci. 2023 Dec 27;15(2):236–44. doi: 10.1021/acschemneuro.3c00409 (PMC10797611; doi:10.1021/acschemneuro.3c00409)
Supplement: Supplementary file 1 — cn3c00409_si_001.pdf [file cn3c00409_si_001.pdf]

Supporting information

A microfluidic high-capacity screening platform for neurological disorders

Lydia Moll[1,2], Johan Pihl[1], Mattias Karlsson[1], Paul Karila[1], Camilla I Svensson[2*]

[1] Cellectricon AB, Mölndal, 431 53, Sweden

[2] Department of Physiology and Pharmacology, Center for Molecular Medicine,
Karolinska Institutet, Stockholm, 171 76, Sweden

Table S1: Primary antibodies

Antibody	Dilution	Cat Nr	Supplier
**Mouse anti-NeuN**	1:1000	ab279296	abcam
**Mouse anti-NeuN**	1:1000	MAB377	Sigma
**Rabbit anti-β-III-tubulin**	1:1000	T2200	Sigma
**Chicken anti-MAP2**	1:5000	ab5392	Abcam
**Guinea pig anti-GFAP**	1:1000	GP52	Progen
**Rabbit anti-Tau [EP2456Y]**	1:1000	ab76128	Abcam
**Rabbit anti-GFAP**	1:1000	70334	Dako
**Chicken anti-beta-III-tubulin**	1:250	AB9354	Sigma

Table S2: Secondary antibodies

Antibody	Dilution	Cat Nr	Supplier
**Goat anti-mouse, Alexa Fluor® 488 plus**	1:1000	A32723	Invitrogen
**Goat anti-mouse, Alexa Fluor 568**	1:1000	A11004	Invitrogen
**Goat anti-mouse, Alexa Fluor 647 Plus**	1:1000	A32728	Invitrogen
**Goat anti-guinea pig, Alexa Fluor 568**	1:1000	A11075	Invitrogen
**Goat anti-rabbit, Alexa Fluor488 plus**	1:1000	A32731	Invitrogen
**Goat anti-rabbit, Alexa Fluor 568**	1:1000	A11036	Invitrogen
**Goat anti-rabbit, Alexa Fluor 647 Plus**	1:1000	A32733	Invitrogen
**Goat anti-chicken, Alexa Fluor 568**	1:1000	A11041	Invitrogen
**Goat anti-chicken, Alexa Fluor 647 plus**	1:1000	A32933	Invitrogen

Table S3: Excitation-emission filter wavelengths

Dye	Microscope	Excitation (nm)	Emission (nm)
**Hoechst**	Operetta	360-400	410-480
	ImageXpress	405 ±20	452 ±45
**Alexa 488**	Operetta	460-490	500-550
	ImageXpress	467.5±21	520±28
**Alexa 568**	Operetta	560-580	590-640
	ImageXpress	555	624±40
**Alexa 647**	Operetta	620-640	650-700
	ImageXpress	638±17	692±40

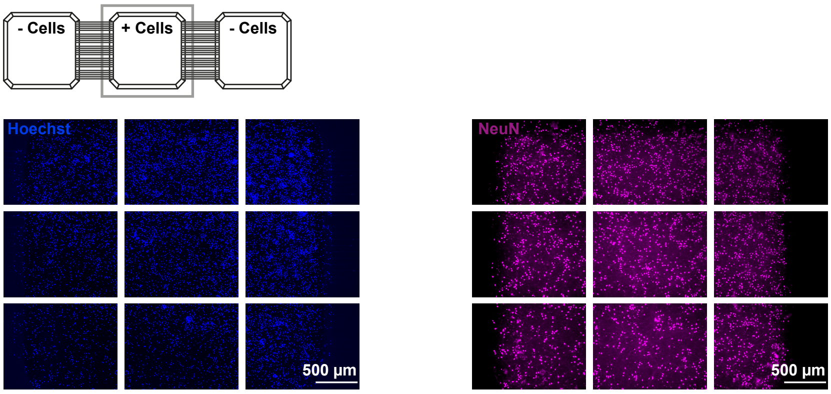

Figure S1: Cells of neuronal cultures (mCtx) at 14 DIV are equally distributed in the well of fusion bonded MC-plates. Images show the same centre well of an experimenal unit (as indicated in the schematic drawing), with nuclei marker Hoechst (left image) and neuronal marker NeuN (right image). Microscope information: Operetta (Perkin Elmer),10x magnification.

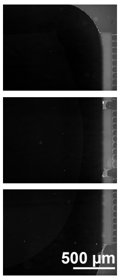

Figure S2: Expanding funnels at the end of each microchannel and the expanded higher box (light grey) before channels are entering the open copartment (black). Microscope information: Operetta (Perkin Elmer), 10x magnification.

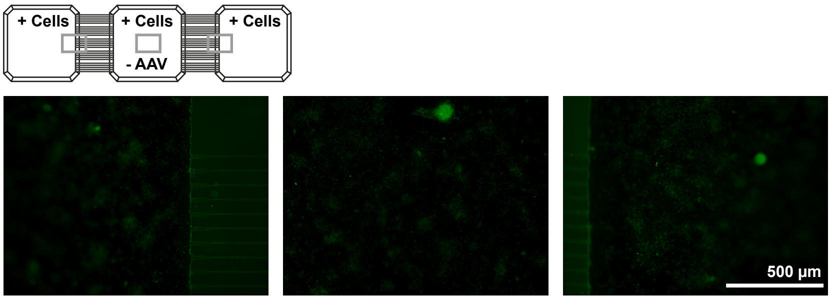

Figure S3: Mouse cortex cells cultured in all three compartments without AAV addition.

A

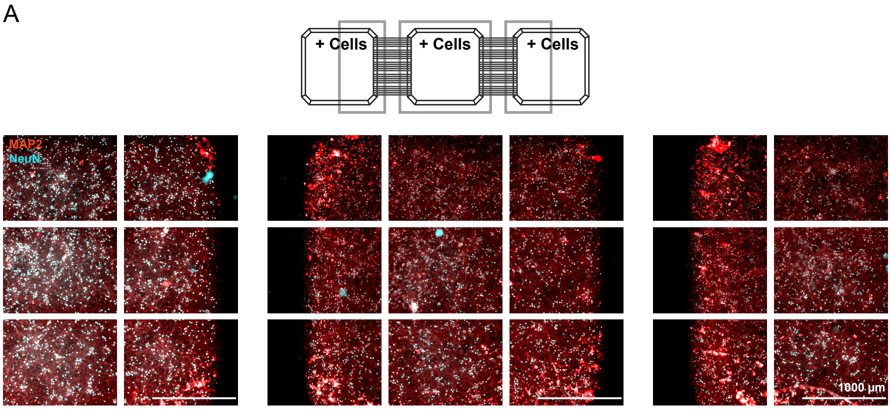

B

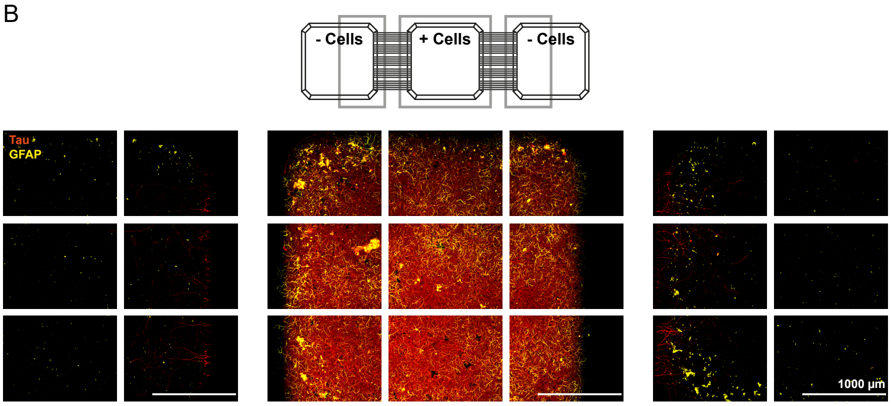

Figure S4: Cells can be seeded in up to 3 connected compartments here examplified in either all three compartments (A) or the center compartment only (B). Microscope information: Operetta (Perkin Elmer), 10x magnification.

A

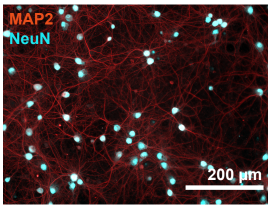

B

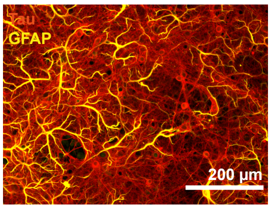

Figure S5: Mouse cortex cultures at 14 DIV in stardard 384 well plates in an excerpt of the well. (A) anti-MAP2 positive dendrites (red) and anti-NeuN stained neuronal cells bodies (cyan); (B) Axons were stained with the marker tau (red) and astrocytes with the glial cell marker GFAP (yellow). Microscope information: Operetta (Perking Elmer), 20x magnification.

A

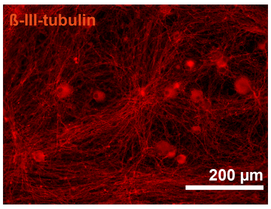

B

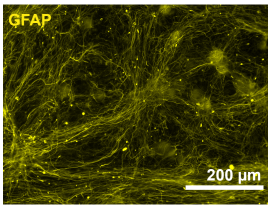

C

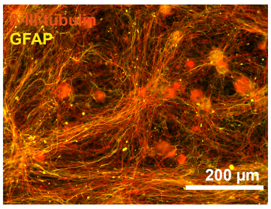

Figure S6: (A) DRG axons immunoreactive to anti ß-III-tubulin (red); (B) glial cells immunoreactive to GFAP (yellow) in an excerpt of a center well at 13 DIV in the MC-plate, as well as an overlay of the same well (C). Microscope information: Operetta (Perkin Elmer), 20x magnification.
